# Cytokeratin7 expression in gastric and colorectal adenocarcinoma: Correlation with prognostic factors

**Published:** 2015

**Authors:** Mohammadreza Jalali-Nadooshan, Sepideh Siadati, Ali Davati, Gholamreza Torabi_Parizi, Samira Ghasemi

**Affiliations:** 1 Depatment of Pathology, Shahed University of Medical Sciences, Tehran, Iran.; 2 Department of Pathology, Babol University of Medical Sciences, Babol, Iran.; 3 Department of Social Medicine and Health, Shahed University of Medical Sciences, Tehran, Iran.; 4 Shahed University of Medical Sciences, Tehran, Iran.

**Keywords:** Cytokeratin 7, Colorectal Cancers, Gastric Cancers, Prognosis.

## Abstract

**Background:**

Gastric and colorectal adenocarcinoma are the second and the fifth most common cancers in Iran, respectively. Expression of cytokeratin 7 (CK7) is established in most malignancies including gastric and colorectal adenocarcinoma. Demonstration of Ck7 could be related to prognostic factors and help to the better management of the patients. The objective of our study was to evaluate the CK7 expression in gastric and colorectal adenocarcinoma and its correlation with other prognostic factors.

**Methods:**

This cross-sectional study was performed on 99 tissue blocks from patients with gastric or colorectal adenocarcinoma undergoing surgery. Tumor grade, tumor size, depth of invasion and metastasis to lymph nodes were determined. Then, the expression of CK7 was studied using immunohistochemistry staining.

**Results:**

Expression of CK7 was 50% and 33.8% in gastric and colorectal adenocarcinoma, respectively. There was not only a significant correlation between CK7 expression and tumor size (r=0.267, P=0.009) but also histologic grade (r=0.222, P=0.028).

**Conclusion:**

CK7 could be more expressed with the increase in tumor size and was associated with poorly differentiated gastric and colorectal adenocarcinoma. However, with these results gathered, it is highly recommended that further studies will be conducted to reveal the exact prognostic role of this factor.

In Iran, adenocarcinoma is the second most common cancer in men (15.93%) and the fourth most common cancer in women (7.73%), while colorectal adenocarcinoma is the fifth most common cancer in men and third in women (1). As we know, determination of prognostic factors help to identify the diagnostic and therapeutic management. Lymph nodes involvement status and depth of tumor invasion are important prognostic factors. Subsequently, biomarkers have been determined as increasingly important in prognosis. One of them are cytokeratins. Cytokeratins (ck) are intermediate cytoskeletal structural proteins in epithelial cells of most organs that play a mechanical supporting role. They are maintained during carcinogenesis (2, 3). Ck7 is one of the low molecular weight cytokeratins and its expression can use to detect the site of origin of metastatic carcinomas. It is expressed in epithelial of many organs, although has little expression in gastric and colon mucosa (3). Cytokeratin genes are the largest group of intermediate fiber complex. Mutation of these genes result to tissue fragility. 

Antibodies against these proteins are used to indicate tissue differentiation and help in pathologic diagnosis (4). The aim of the current study was to evaluate the CK7 expression in gastric and colorectal adenocarcinoma as well as its correlation with some prognostic factors.

## Methods

Ninety-nine paraffin-embedded tissue blocks were obtained from patients with gastric and colorectal adenocarcinoma who had undergone gastrectomy or colectomy in Mostafa Khomeini Hospital, Tehran, Iran. Data including tumor size (measured in cm), tumor grade (well, moderate and poorly differentiated), depth of tumor invasion and lymph node status were retrieved from the files maintained at pathology department. 

Tissue blocks were cut consecutively at 3 microns. One slide from each block was stained with conventional H and E method. Immunohistochemistry staining for CK7 was performed on another slide using the monoclonal antibody according to manufacturer’s instruction. (novocastro, UK). The presence of CK7 was scored under high power (400x) in 1000 tumor cells and the percentage of positive inmunostaining (5%) was determined. 

Statistical analysis was performed using SPSS 17 software, t-test, Mann-Whitney, chi-square and Spearman correlation tests. A p-value of <0.05 was considered statistically significant.

## Results

A total of 99 gastric and colorectal adenocarcinoma were retrieved from pathology department and evaluated for CK7 immunostaining. Table 1 revealed demographic characteristics of patients and pathologic findings of tumors. In our study, gastric carcinoma was more common in women in comparision to men. Also, gastric carcinoma had worse grade than colorectal carcinoma.

Spearman correlation between percentage of CK7 positive cells and prognostic factors are shown in table 2. For some prognostic factors (tumor size and tumor grade), this correlation was significant (based on Mann-Whitney 0.036) (table 3). 

Considering 5% CK7 stained cells as positive, the relation was found between CK7 and some prognostic factors.

**Table 1:** Demographic characteristic of patients and pathologic findings of tumors

**Tumor characters**	**Stomach**	**Colorectal**	**P-value**
Tumor origin	34 (34.3%)	65 (65.7%)	0.002
**Gender** Man Woman	27 (79.4%) 7 (20.6%)	34 (52.3%) 31 (47.7%)	0.008
Age (mean±SD)	63±11.7	59.6±15.1	0.246
Tumor size	5.76±2.5	4.85±2.22	0.072
**Tumor grade** Well Moderate Poor	7 (20.6%) 14 (41.2%) 13 (38.2%)	44 (67.7%) 14 (21.5%) 7 (10.8%)	< 0.001
Full-thickness involvement	88.2%	86.2%	0.771
Lymph node involvement	58.8%	44.6%	0.179
Cytokeratin positive cells	51.5%	33.8%	0.09

**Table 2:** Spearman correlation between percentage of cytokeratin positive cells and prognostic factors

**Tumor charactors**	**Total**	**Stomach**	**Colorectal**
Tumor grade	r=0.222 P=0.028	r=0.292 P=0.099	r=0.056 P=0.656
Tumor size	r=0.267 P=0.009	r=0.359 P=0.044	r=0.157 P=0.215
Depth of tumor	r=0.121 P=0.234	r=0.110 P=0.541	r=0.106 P=0.402
Number of involved lymph node	r=0.132 P=0.192	r=0.033 P=0.853	r=0.161 P=0.200

**Table 3:** Expression of CK7 in different grades of gastric and colorectal adenocarcinoma based on Mann-Whitney 0.036

**Grade of Tumor**	**Expression of cytokeratin 7**	
	**Negative**	**Positive**
Well differentiated	35(68.6%)	16(31.4%)
Moderately differentiated	16(59.3%)	11(40.7%)
Poorly differentiated	8(40.0%)	12(60.0%)
Total	59(60.2%)	39(39.8%)

## Discussion

This study was performed on 99 tissue blocks from patients with gastric and colorectal adenocarcinoma, in which there was correlation between CK7 expression and tumor size and also histologic grade. Ck7 is cytoplasmic filaments of epithelial cells. With respect to specific CK profile of carcinoma, we could find the relation of the Ck7 expression and other prognostic factors.

Demonstration of prognostic factors is used to determine the best therapeutic management. Previous studies showed CK7 expression in a few colorectal and gastric adenocarcinoma. We could not find any study with emphasis on the importance of CK7 expression in these tumors. As previously mentioned, the size of gastric and colorectal tumors had correlation with positive CK7 staining. On the other hand, in tumor, node, metastasis (TNM) system, tumor size of this type of carcinoma has no prognostic importance. Therefore, the correlation of CK7 and tumor size do not necessarily mean a poor prognostic indication. However, this correlation does not hold true for gastric and colorectal adenocarcinoma separately. Although this may be a result of small sample size.

Chu et al. stained 435 cases of epithelial malignancy using CK7 and CK20 immunohistochemistry staining, considering 5% stained cells as positive score. One hundred percent of lung, ovary, uterus, and salivary gland cancers were CK7 positive. Malignancy of stomach (38%), esophagus (21%), kidney (11%) and colorectal (5%) were at the bottom list (5). In our study, we evaluated the percentage of positive cells beside the 5% cutoff point. We found the frequencies of CK7 expression (with 5% score) in gastric and colorectal adenocarcinoma to be 50% and 33.8%, respectively. Again, there was a correlation between the percentage of stained cells and tumor size. Although the difference could be due to the small number of cases in their study (only 8 cases of stomach and 20 cases of colon).

Poorly differentiated grade of the tumor is a poor prognostic factor and does not have clinically importance in therapeutic management. But the determination of tumor differentiation can assist to recognize the pathogenecity of adenocarcinomas. Radovic et al. found that CK7 was not found in normal colonic mucosa, although it was found in a few cases with inflamed and degenerative mucosa. Interestingly, this marker was found in all dysplastic cells with no correlation with the severity of dysplasia. Also, some adenocarcinoma was CK7 positive. Therefore, dysplastic lesions of colorectal mucosa are apparently CK7 positive. With progression to carcinoma, this marker disappeared again (6). In our study, we noticed that a decrease in tumor cell differentiation resulted in the CK7 expression and increased percentage of the stained cells. This finding might indicate the reexpression of CK7 in carcinogenesis. Whereas, this is totally different in the stomach. Although rare, expression of CK7 in non-malignant mucosa of stomach is probable. Gastric surface epithelial and glandular cells usually express CK7 (7). Based on Oksanen *et al*. CK7 expression had no association with H-pylori (8). Consequently, there is little possibility that marker appeared in the process of tumor growth. In our study, no correlation was found between the depth of tumor invasion and CK7. Considering that most of our cases were in T3 stage (of TNM), our analytical precision may be compromised. 

In our study, no significant correlation was found between the lymph nodes involvement and CK7 expression. Bayrak et al. studied 196 cases of colorectal adenocarcinoma and showed that Ck7 positivity had association with lymph node metastasis (3). This difference may be due to sample size. Based on the above findings, we recommend to evaluate CK7 expression in pelvic tumors with caution. CK7 and CK20 profile analysis is one of the methodologies that was used for the determination of ovarian tumor origin. If CK7 was positive, most likely it is ovarian tumor (9-11). Some ovarian metastases from gastric and colorectal adenocarcinoma and the increase in the size of these tumors result in the increase of CK7 expression. Therefore, CK7 expression cannot exclude metastasis from gastrointestinal adenocarcinoma.

Also, Kummar et al. found similar results. They evaluated the negative expression of CK7 in the diagnosis of metastasis lung tumors to colon and considering that CK7 expression could be the indication of lung origin. In their study, 2 out of 24 metastatic cases were positive for CK7 (12). Again, considering the lack of large number of these cases, one must be cautious with these results. 

**Conclusion: **CK7 seems to be positive with the increase in tumor size and the grade of gastric and colorectal adenocarcinoma associated with poor prognosis. In the course of colorectal adenocarcinoma, this marker may be reproduced. Considering these findings and a few studies performed in this field, further studies with more cases are needed to be followed-up to reveal the exact prognostic role of this factor.

## Notes

**Funding: **This paper resulted from a medical student thesis and was ﬁnancially supported by Shahed University, Tehran, Iran.

## References

[1] Kolahdoozan S, Sajadi A, Radmard AR, Khademi H (2010). Five common cancers in Iran. Arch Iran Med.

[2] MeSH Browser [database on the Internet] (2009). Bethesda (MD): National Library of Medicine (US); 2009.

[3] Bayrak R, Yenidunya S, Haltes H (2011). Cytokeratin 7 and Cytokeratin 20 expression in colorectal adenocarcinoma. Pathol Res Pract.

[4] Schweizer J, Bowden PE, Coulombe PA, Langbein L, Lane EB, Magin ThM (2006). New consensus nomenclature for mammalian keratins. J Cell Biol.

[5] Chu P, Wu E, Weiss LM (2000). Cytokeratin 7 and cytokeratin 20 expression in epithelial neoplasms: a survey of 435 cases. Mod Pathol.

[6] Radovic S, Selak I, Babic M, Vakobrat-Bijedic Z, Knezevic Z (2004). Anti-cytokeratin 7: a positive marker for epithelial dysplasia in flat bowel mucosa. Bosn J Basic Med Sci.

[7] Gurzu S, Jung I (2012). Aberrant pattern of the cytokeratin 7/cytokeratin 20 immunophenotype in colorectal adenocarcinomas with BRAF mutation. Pathol Res Pract.

[8] Oksanen A, Sankila A, Von Boguslawski K, Sipponen P, Rautelin H (2005). Inflammation and cytokeratin 7/20 staining of cardiac mucosa in young patients with and without Helicobacter pylori infection. J Clin Pathol.

[9] Ramalingam P, Malpica A, Silva EG (2004). The use of cytokeratin 7 and EMA in differentiating ovarian yolk sac tumors from endometrioid and clear cell carcinomas. Am J Surg Pathol.

[10] Dai L, Song Q, Li L, Zhong D, Hui Y (2001). Expression of cytokeratin 7 and 20 in ovarian metastatic carcinomas. Zhonghua Bing Li Xue Za Zhi.

[11] Cathro HP, Stoler MH (2002). Expression of cytokeratins 7 and 20 in ovarian neoplasia. Am J Clin Pathol.

[12] Kummar S, Fogarasi M, Canova A, Mota A, Ciesielski T (2002). Cytokeratin 7 and 20 staining for the diagnosis of lung and colorectal adenocarcinoma. Br J Cancer.

